# A Case of Lung Squamous Cell Carcinoma Harboring TP53 Mutation and PLPP5‐FGFR1 Fusion Gene

**DOI:** 10.1111/crj.70074

**Published:** 2025-04-17

**Authors:** Meng Xiao‐ru, Shi Xiao‐Xiong, Gao Qian, Qu Ya‐jing, Huo Li‐li, Gao Yan‐Yan, Xu Peng‐peng, Ma Guan‐nan, Ren Gui‐bing

**Affiliations:** ^1^ Oncology Department Characteristic Medical Center of Chinese People's Armed Police Force Tianjin China; ^2^ Medical Research Center Key Laboratory of Digital Technology in Medical Diagnostics of Zhejiang Province Hangzhou China

**Keywords:** lung squamous cell carcinoma, PLPP5‐FGFR1 fusion gene, TP53 mutation

## Abstract

Lung squamous cell carcinoma (LUSC) is one of the most common subtype of lung cancer and is associated with the poor prognoses. The fibroblast growth factor receptor (FGFR) family is known to be activated through fusions with various partners across multiple cancer types, including nonsmall cell lung cancer (NSCLC). FGFR inhibitors are currently undergoing clinical evaluation for the treatment of tumors harboring these fusions. While FGFR1 amplification has been well‐documented in numerous NSCLC datasets, the characterization of specific FGFR fusion variants remains limited. In this study, we identified a novel PLPP5‐FGFR1 fusion in a 65‐year‐old male patient with lung squamous cell carcinoma through targeted RNA sequencing. The fusion junction was located between exon 1 of PLPP5 and exon 5 of FGFR1, and the result was validated by Sanger sequencing. To our knowledge, this is the first reported case of a PLPP5‐FGFR1 fusion coexisting with a TP53 mutation in LUSC. These findings broaden the spectrum of potential translocation partners in FGFR1 fusions, and the clinical implications of this novel fusion on treatment outcomes and prognosis warrant further investigation and long‐term follow‐up.

## Graphical abstract

Lung squamous cell carcinoma (LUSC) is a common cancer with poor prognosis. FGFR fusions, including in nonsmall cell lung cancer (NSCLC), are targeted by FGFR inhibitors. This study reports a novel PLPP5‐FGFR1 fusion in a 65‐year‐old LUSC patient, coexisting with a TP53 mutation. These findings expand FGFR fusion possibilities and highlight the need for further clinical investigation into treatment and prognosis impacts.

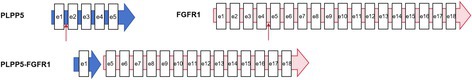

## 1 Introduction

Lung cancer is one of the leading causes of cancer‐related morbidity and mortality worldwide, with lung squamous cell carcinoma (LUSC) being particularly prevalent among smokers [1]. The molecular mechanisms underlying LUSC are complex and involve various genetic alterations, with fusion genes playing a crucial role as key drivers in tumor initiation and progression. Fusion genes occur when two originally separate genes are joined through chromosomal rearrangements or genetic mutations, potentially resulting in the production of abnormal proteins or altering protein function. Although research on LUSC is less extensive compared to lung adenocarcinoma, certain fusion genes have been identified as being associated with the onset, progression, and prognosis of LUSC [2]. These fusion genes promote tumor progression through mechanisms such as enhancing cell proliferation, modulating cell migration, interfering with DNA repair, and facilitating immune evasion [3, 4].

Fibroblast growth factor receptors (FGFRs) are a family of transmembrane receptor tyrosine kinases (RTKs). FGFR gene fusions can activate the kinase domain, contributing to the initiation and progression of various cancers [5, 6, 7]. Small molecule inhibitors targeting FGFR fusion genes have demonstrated significant tumor‐suppressive effects. While FGFR1 fusion events are relatively rare, their primary oncogenic mechanism is typically overexpression [8, 9, 10].

PLPP5 encodes a phosphatase located in the chromosomal region 8p11–12, which is frequently amplified in various epithelial cancers [11]. It is closely associated with cancer cell survival and transformation, particularly in breast cancer, pancreatic cancer, and lung cancer, where it is considered a key driver of tumorigenesis [12, 13]. The amplification and overexpression of PLPP5 are linked to enhanced tumor survival and growth. Additionally, PLPP5 may play a role in the development, maturation, and differentiation of B lymphocytes, as well as antibody production by plasma cells [14]. Consequently, its fusion with FGFR1 may influence immune responses, as well as the proliferation, migration, and survival of tumor cells. Such a fusion event could lead to the overexpression or activation of FGFR1, thereby promoting tumor progression.

Understanding the role of genetic alterations, including TP53 mutations and gene fusions, is crucial for the development of targeted therapies and personalized treatment strategies. While FGFR1 amplification has been well‐defined across multiple nonsmall cell lung cancer (NSCLC) datasets, the various FGFR fusion variants remain under‐characterized [15]. In this study, we report a novel case of a PLPP5‐FGFR1 fusion coexisting with a TP53 mutation in a patient with LUSC. We discuss the potential implications of this novel fusion for targeted therapies, clinical outcomes, and personalized treatment approaches, detailing the patient's clinical presentation, pathological findings, diagnostic process, treatment course, and the impact of this fusion on treatment responses.

## 2 Clinical and Pathological Characteristics

### 2.1 Case Summary

The patient is a 65‐year‐old Chinese male with a long history of smoking and alcohol consumption, but no history of exposure to chemical, radioactive substances, or toxins. He has no significant medical history, including hypertension, coronary heart disease, diabetes, or other notable illnesses, and there are no hereditary diseases in his family.

On October 20, 2023, the patient was admitted to the hospital due to the “discovery of a lung mass for over a month.” Imaging studies revealed a mass in the left upper lobe bronchus, left hilar lymphadenopathy, and multiple small nodules in both lungs. A PET‐CT scan indicated a hypermetabolic lesion in the left upper lung hilum, suggestive of central lung cancer. Tumor markers showed elevated ferritin and keratin fragments. A bronchoscopic biopsy confirmed the diagnosis of moderately differentiated squamous cell carcinoma. From November 2023 to January 2024, the patient was hospitalized several times for neoadjuvant chemotherapy and immunochemotherapy. Antineoplastic protocols included teriprizumab, liposomal paclitaxel, and cisplatin. On January 20, 2024, the patient underwent a left upper lobectomy, pulmonary artery plasty, and mediastinal lymph node dissection under general anesthesia. Postoperative pathology confirmed moderately differentiated squamous cell carcinoma, with tumor invasion into the adventitia of the pulmonary artery and metastasis to lymph nodes in the 12th group. Sequencing of the lung biopsy specimen revealed a TP53 mutation coexisting with PLPP5‐FGFR1 gene fusion. From February to May 2024, the patient was hospitalized multiple times for ongoing immunochemotherapy (including teriprizumab, liposomal paclitaxel, and cisplatin) and symptomatic treatment, including anti‐inflammatory therapy, liver protection, and correction of anemia. The patient has been followed up for 6 months with ongoing clinical monitoring.

### 2.2 Pathological Examination

**FIGURE 1:**
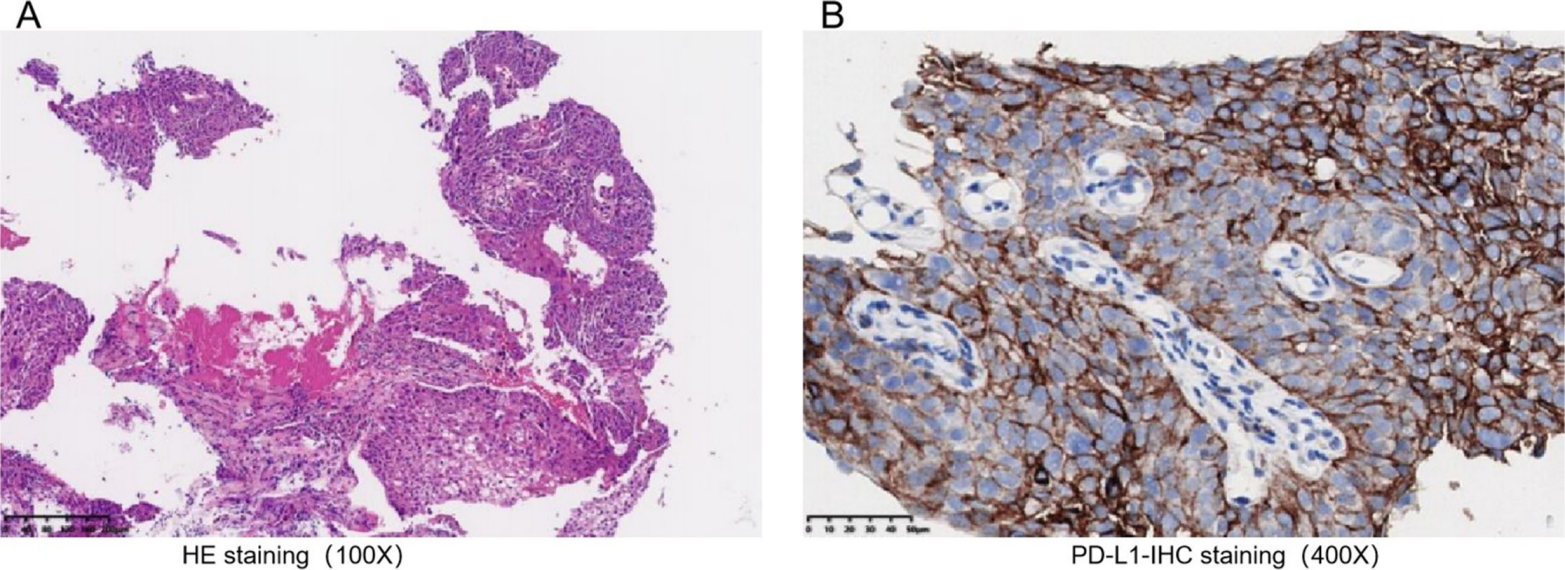
Pathological morphological features and IHC results of the left upper lung lobe biopsy specimen from the patient on November 3, 2023. (A) The biopsy result of the left upper lung lesion indicates squamous cell carcinoma. HE staining, ×100. (B) PD‐L1 IHC staining of the left upper lung lesion, ×400. HE, hematoxylin and eosin; IHC, immunohistochemistry; PD‐L1, programmed death‐ligand 1.

Bronchoscopic biopsy confirmed the presence of moderately differentiated squamous cell carcinoma. PD‐L1 immunohistochemistry revealed a tumor proportion score (TPS) of 65% and a combined positive score (CPS) of 70, indicating PD‐L1 positivity (Figure 1A,B). Postoperative pathology showed tumor invasion into the adventitia of the pulmonary artery and confirmed metastasis to the 12th group of lymph nodes (2/2 positive).

### 2.3 Fusion Description

**FIGURE 2:**
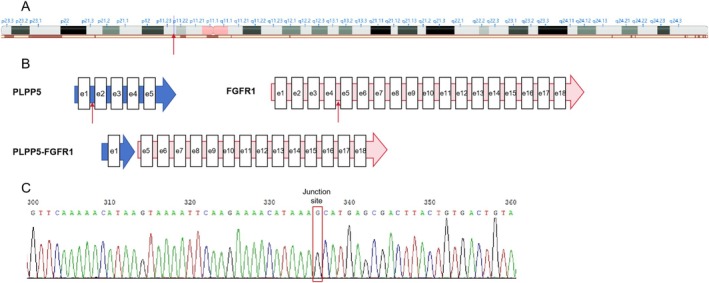
Identification of a novel PLPP5‐FGFR1 fusion. (A) The chromosomal locations of the fusion breakpoints for the FGFR1 and PLPP5 genes. (B) Schematic representation of the structures of PLPP5, FGFR1, and the PLPP5‐FGFR1 fusion transcript. (C) Sanger sequencing confirmed the fusion of exon 1 of the PLPP5 gene with exon 5 of the FGFR1 gene.

Fusion gene testing was performed on the lung biopsy specimen using RNA sequencing. Molecular analysis revealed a novel fusion transcript between exon 1 of PLPP5 and exon 5 of FGFR1 (Figure 2A–D). The PLPP5‐FGFR1 gene fusion was subsequently confirmed through Sanger sequencing, which verified the fusion between exon 1 of the PLPP5 gene on chromosome 8 and exon 5 of the FGFR1 gene (Figure 2E).

**FIGURE 3:**
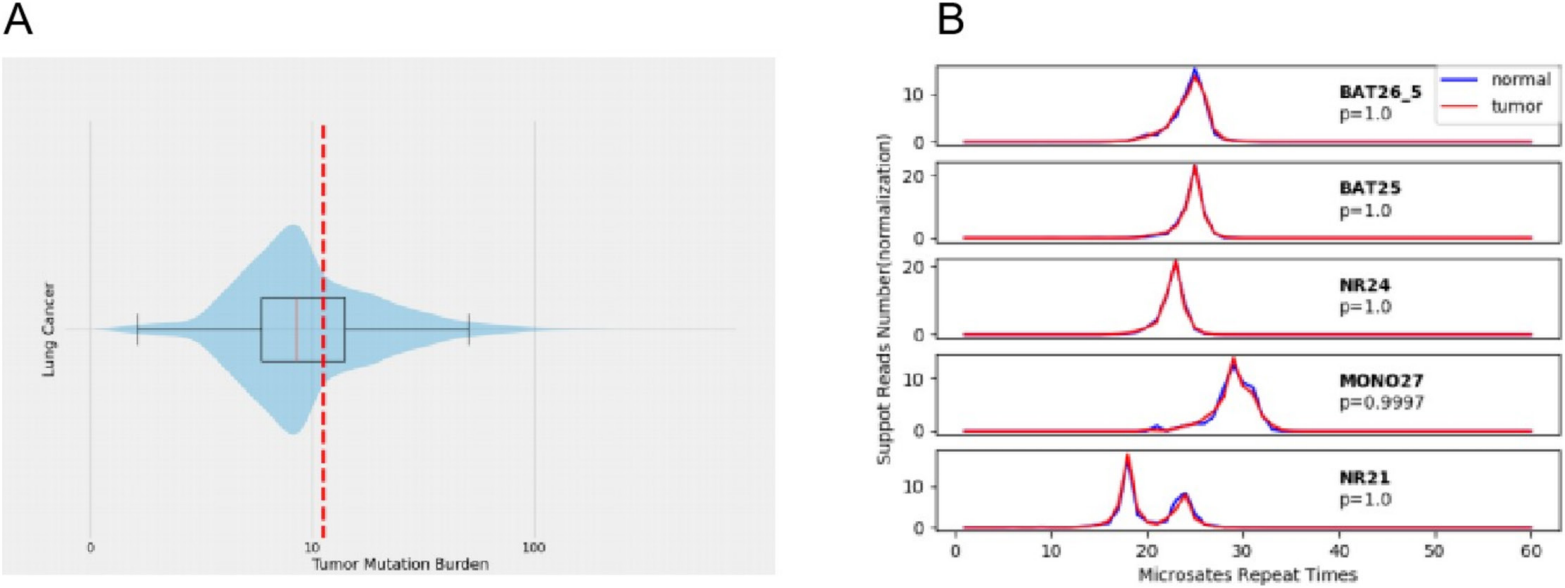
Tumor mutation burden and microsatellite instability. (A) Tumor mutation burden. (B) Microsatellite instability.

Additionally, the tumor mutation burden (TMB) was calculated based on the number of nonsynonymous mutations per million bases (Mb) from targeted sequencing. The TMB was found to be 11.27 mutations/Mb (Figure 3A). Furthermore, microsatellite analysis revealed 105 detected microsatellites, with a microsatellite instability (MSI) score of 0.124, indicating no significant microsatellite instability in the tumor cells (Figure 3B).

## 3 Diagnosis and Treatment

The final diagnosis was central lung cancer (squamous cell carcinoma) complicated by obstructive pneumonia and atelectasis. The treatment regimen included multiple cycles of chemotherapy (liposomal paclitaxel and cisplatin), immunotherapy (teriprizumab), and surgical resection. Postoperative recovery was favorable, and the patient's condition remains stable.

## 4 Discussion

In this report, we identified a novel PLPP5‐FGFR1 fusion and TP53 p.H179Y mutation in a 65‐year‐old male patient with lung squamous cell carcinoma (LUSC) through genetic testing of the tumor. To our knowledge, this is the first reported case of a PLPP5‐FGFR1 fusion with TP53 mutation in lung cancer or any other cancer type. These findings expand the spectrum of translocation partners associated with FGFR1 fusions. The fusion was further confirmed through Sanger sequencing. This discovery provides valuable insight into the selection of FGFR1 fusion detection methods, which could facilitate more accurate diagnosis and targeted treatment strategies.

Fibroblast growth factor receptors (FGFRs) are a family of transmembrane receptor tyrosine kinases (RTKs) that play critical roles in various cell signaling pathways. FGFR gene fusions can result in abnormal activation of the kinase domain, driving the initiation and progression of multiple cancers [16, 17]. PLPP5 encodes a phosphatase which is frequently amplified in epithelial cancers, including breast, pancreatic, and lung cancers [11, 12]. PLPP5 is considered a key driver of tumor cell survival and transformation [6]. The novel PLPP5‐FGFR1 fusion identified in this case may contribute to the amplification of FGFR1 and could potentially be targeted with FGFR inhibitors, especially in recurrent tumors.

This study represents the first report of the PLPP5‐FGFR1 fusion event, marking a new form of genomic alteration involving FGFR1. In addition to the fusion, the patient also exhibited a TP53 p.H179Y mutation, located in exon 5 of the TP53 gene [18]. This mutation is situated within the DNA‐binding domain of the TP53 protein and is associated with impaired transcriptional activation, reduced proliferative suppression, and decreased Nrf2 expression, all of which may promote tumor cell proliferation [19]. The combined effects of FGFR fusion and TP53 mutations could act synergistically in tumor initiation and progression. TP53 mutations compromise DNA repair mechanisms, increasing genomic instability, such as chromosomal translocations [20], which may, in turn, facilitate the formation of FGFR fusions. This genomic instability could exacerbate tumor malignancy. Additionally, TP53 mutations drive uncontrolled cell cycle progression [21], while FGFR fusion genes activate signaling pathways such as MAPK and PI3K [22, 23]. The interaction between TP53 mutations and FGFR fusions may further promote cell proliferation, survival, and migration, potentially enhancing the malignancy of the tumor.

The patient has been followed for 6 months, with no evidence of recurrence or metastasis to date. During follow‐up, we found that this patient with a PLPP5 fusion mutation and a TP53 mutation developed immune‐related nephritis and anemia on day 38 of neoadjuvant therapy, and immune‐related pneumonia a year after surgery. Clinically, he seemed more prone to immune‐related adverse events than other lung squamous cell carcinoma patients. Moreover, he had persistent mild‐to‐moderate anemia that was difficult to correct. While managing these adverse reactions, we also considered the broader implications of the PLPP5‐FGFR1 fusion. The long‐term impact of this genetic rearrangement on treatment and prognosis requires further follow‐up. To our knowledge, this is the first reported instance of the PLPP5‐FGFR1 fusion in lung cancer or any other malignancy. This rarity could stem from the low incidence of FGFR1 fusions in common tumors and limited detection methods. Though the clinical significance of such fusions remains uncertain, precedents in other cancers—such as FGFR2 fusions showing therapeutic potential in cholangiocarcinoma patients treated with pemigatinib [24] and futibatinib [25]—suggest that FGFR alterations may play a significant role in targeted therapy and prognosis in lung squamous cell carcinoma, warranting further investigation.

### 4.1 Patient Perspective

This study was reviewed and approved by the Characteristic Medical Center of Chinese People's Armed Police Force (No. 2025‐0001). The publication of this paper has been approved by the authors through informed consent.

### 4.2 Materials and Methods

#### 4.2.1 Targeted RNA Sequencing and Gene Fusion Identification

A fusion gene panel was used to target 22 frequently rearranged genes, including ALK, FGFR1, ROS1, BRAF, FGFR2, TMPRSS2, BRCA1, FGFR3, BRCA2, MYB, CD74, NRG1, EGFR, NTRK1, ETV4, NTRK2, ETV5, NTRK3, ETV6, PDGFRA, EWSR1, and RET. Nucleic acid extraction, library preparation, hybrid capture, and next‐generation sequencing (NGS) were performed using the Illumina NextSeq 500 platform (San Diego, CA) at the Key Laboratory of Medical Diagnosis Digital Technology, Zhejiang Province. FASTQ sequencing files were aligned to the human reference genome (UCSC hg19) using BWA software. Gene fusions were identified using STAR‐Fusion and Arriba software.

#### 4.2.2 Sanger Sequencing

Direct Sanger sequencing was performed on PCR products to confirm the NTRK3 fusion. The following primers were used to amplify the NTRK3‐AJUBA fusion transcript:forward primer:CTGAGCCCAAGCCTCTCAAA and reverse primer:CACTGGCAAGGTAAGCAAAGT.

#### 4.2.3 PD‐L1 Immunohistochemical Detection

PD‐L1 expression was assessed using the PD‐L1 antibody with clone number 22C3. The positive expression rate was determined by calculating the tumor proportion score (TPS) and the combined positive score (CPS), which were used to evaluate PD‐L1 expression levels.

## Author Contributions

Ren Gui‐bing and Ma Guan‐nan conceived and designed the study. Meng Xiao‐ru, Shi Xiao‐Xiong, and Gao Qian collected the data and performed the analysis. All authors contributed to the interpretation of the data and writing of the manuscript, and approved the final version.

## Conflicts of Interest

The authors declare no conflicts of interest.

## Data Availability

The data that support the findings of this study are available from the corresponding author upon reasonable request.
